# Antiviral, Antioxidant, and Antihemolytic Effect of *Annona muricata* L. Leaves Extracts

**DOI:** 10.3390/plants9121650

**Published:** 2020-11-26

**Authors:** Ana Paola Balderrama-Carmona, Norma Patricia Silva-Beltrán, Juan-Carlos Gálvez-Ruiz, Saúl Ruíz-Cruz, Cristóbal Chaidez-Quiroz, Edgar Felipe Morán-Palacio

**Affiliations:** 1 Departamento de Ciencias Químico Biológicas y Agropecuarias, Universidad de Sonora, Unidad Regional Sur, Navojoa, Sonora 85880, Mexico; paola.balderrama@unison.mx (A.P.B.-C.); edgar.moran@unison.mx (E.F.M.-P.); 2 Departamento de Ciencias de la Salud, Campus Cajeme, Universidad de Sonora, Ejido Providencia, Cd, Obregón, Sonora 85050, Mexico; 3 Departamento de Ciencias Químicas Biológicas, Universidad de Sonora, Hermosillo, Sonora 83000, Mexico; juan.galvez@unison.mx; 4 Departamento de Biotecnología y Ciencias Alimentarias, Instituto Tecnológico de Sonora, Cajeme, Sonora 85000, Mexico; saul.ruiz@itson.edu.mx; 5 Centro de Investigación en Alimentación y Desarrollo A.C., Laboratorio Nacional para la Investigación en Inocuidad Alimentaria, Culiacán, Sinaloa 80110, Mexico; chaqui@ciad.mx

**Keywords:** UPLC, bacteriophages, antiviral, antioxidant capacity, rutin, soursop

## Abstract

*Annona muricata* L. is a tropical tree that is used in traditional medicine around the world. The high content of flavonoid, alkaloid, acetogenin, phenolic and lipophilic compounds of this tropical tree forms the basis of its traditional medical uses. Our objective was to study soursop leaf extracts to support their use as antiviral therapies and investigate their protective effects against oxidative damage. The aqueous extract (AE) and acidified ethanolic extract (AEE) of soursop leaves were characterized by ultra performance liquid chromatography (UPLC), and their effects on human erythrocytes and in vitro antioxidant capacity, as evaluated by 2,2-diphenyl-1-picrylhydrazyl (DPPH) and 2,2′-azino-bis (3-ethylbenzothiazoline-6-sulfonic acid (ABTS) assays, were investigated. The antiviral effects were evaluated using a bacteriophage surrogate. AEE showed the highest phenolic content, with rutin as the predominant compound. This extract showed higher values in the DPPH and ABTS assays, with 23.61 ± 0.42 and 24.91 ± 0.16 mmol of Trolox equivalent per gram, respectively. Inhibition of hemolysis was 34% and 51% for AE and AEE, respectively. AEE was selected for the antiviral study because of its higher antioxidant activity. The viral reduction ranged from 5–6 log10 plaque-forming units/volume (PFU) at contact times of 15–360 min. Soursop leaves have a positive effect on reducing oxidative stress in human erythrocytes and viral infections.

## 1. Introduction

*Annona muricata* Linn. (Annonaceae) is commonly known as “Soursop” or “Graviola.” It is a terrestrial deciduous tree and produces an edible fruit. This species of *Annona* has been grouped with the “cherimoya” plants of the Annonaceae family. Although this Annonacea is native to America, it has now become established in many tropical countries. The (*Annona muricata* L.) is a plant species used in Mexican traditional medicine, where the leaves are used to treat various diseases, such as stomach pain, bronchitis and gastric cancer, and it has even been called a “cancer killer” [1,2]. In Africa, Asia and South America, the seeds of this species of *Annona* are used to treat several types of cancer due to the chemopreventive properties presented by *A. muricata* extracts, which can induce apoptosis of cancer cells [3], along with the synergistic effects of polyphenols, flavonoids, alkaloids and lipophilic antioxidant compounds [4]. Also, it is well known that the species of the Annonaceae family besides producing polyphenols, flavonoids and alkaloids, produces large quantities of acetogenins, a secondary metabolite derived from long-chain fatty acids [3,4] that have been found to have anticancer, anti-inflammatory, antibacterial and antiviral properties [3,5]. The phenolic compounds in *A. muricata*, such as quercetin and gallic acid, are reported to be the compounds most responsible for the antioxidant capacity of the plant [5]. However, the ability of soursop leaf extracts to inhibit the generation of free radicals has not been tested in human erythrocytes, and studies have reported that at least 100 human diseases, including hypertension, diabetes, renal insufficiency, Parkinson’s disease and Alzheimer’s disease, are related to cellular oxidative damage [6]. On the other hand, *A. muricata* extracts have shown effectiveness against viruses because they can decrease viral replication [7]. Nonetheless, to our knowledge, no evidence of the antiviral capacity of soursop leaves on enteroviruses has been reported. Bacteriophages have been used as indicators or surrogates for human pathogenic viruses due to their similarities in morphology and resistance to external factors, together with their safety for humans and easy handling in the laboratory [8]. The in vitro assay used to assess the lytic properties of bacteriophages is a simple, rapid, inexpensive, sensitive and highly specific method [9]. Bacteriophages Av-05 and Av-08 have been characterized by Amarillas et al. [10] and Lopez-Cuevas et al. [11], who reported that their structural and genetic characteristics are similar to enteric viruses and, therefore, represent a perfect enterovirus model. The present research was developed to further study the biological properties of *A. muricata* L. leaves, characterizing phenolic compounds by ultra performance liquid chromatography (UPLC), and evaluating their antioxidant effects (inhibition of hemolysis) on human erythrocytes and their antiviral effectiveness by using bacteriophages as a human viral surrogate.

## 2. Results

### 2.1. UPLC Analysis

Analysis of the phenolic content was carried out to establish a relationship with antioxidant and antiviral potential. The results are shown in Table 1. 

On the other hand, the values of the chromatographic method validation parameters such (chromatograms, absorption spectra, linearity and detection) are shown in the Supplementary Material.

The profile of phenolic compounds of acidified ethanolic extract (AEE) shows the presence of two predominant compounds, the flavonoids rutin (quercetin 3-*O*-rutinoside; 2) and naringenin (5,7-dihydroxy-2-(4-hydroxyphenyl) chroman-4-one; 3); one phenolic aldehyde, vanillin (4-hydroxy-3-methoxybenzaldehyde; 4); one hydroxybenzoic acid, gallic acid (3,4,5-trihydroxybenzoic acid; 1); and one allylbenzene, eugenol (4-allyl-2-methoxyphenol; 5), presented in decreasing order of concentration of their major constituents. In contrast, aqueous extract (AE) only showed the presence of rutin. The compounds found in the highest concentrations in AEE were rutin and naringenin, which showed levels of 6.52 ± 0.59 and 5.22 ± 0.75 mg/g extract of dry weight. On the other hand, AE showed a low concentration of rutin of 1.20 ± 0.06 mg/g. Other compounds detected in AEE were gallic acid (3.13 ± 0.14 mg/g), vanillin (3.60 ± 0.17 mg/g) and eugenol (1.40 ± 0.04 mg/g).

### 2.2. DPPH and ABTS Assays

The antioxidant activity of the soursop leaf extracts measured by the 2,2-diphenyl-1-picrylhydrazyl (DPPH) assay is shown in Table 2. It was observed that AEE neutralized the DPPH radical, showing inhibition values of 23.61 ± 0.42 mmol TE/g. In contrast, AE had a lower antioxidant capacity, reaching an inhibition value of only 2.97 ± 0.40 mmol TE/g. Significant differences were observed between treatments (*p* ≤ 0.05). However, when the antioxidant activity of the leaf extracts was measured by the 2,2′-azino-bis (3-ethylbenzothiazoline-6-sulfonic acid) (ABTS) test, it could be observed that AEE possessed a greater capacity to neutralize the ABTS radical, showing an inhibition value of 24.91 ± 0.16 mmol TE/g. A lower antioxidant capacity was presented by AE, with an inhibition value of 17.9 ± 0.31 mmol TE/g. Significant differences were observed between extracts.

### 2.3. Hemolysis Assays in Human Erythrocytes

To determine the protective capacity of *A. muricata* L. leaf extracts, lysis was induced in human erythrocytes using 2-2′-azobis (2-amidinopropane) dihydrochloride (AAPH), which acts by oxidizing lipids and membrane proteins, leading to hemolysis [12]. It was observed that the AEE had a more significant protective effect against oxidative damage, with 51.21 ± 0.36% inhibition of hemolysis. In contrast, the aqueous extract showed lower protection against oxidative stress, presenting a hemolysis inhibition value of 34.16 ± 0.13% (Table 3).

### 2.4. Antiviral Assay

As AEE showed the highest antioxidant capacity, this extract was evaluated for its antiviral effect. 

**Figure 1:**
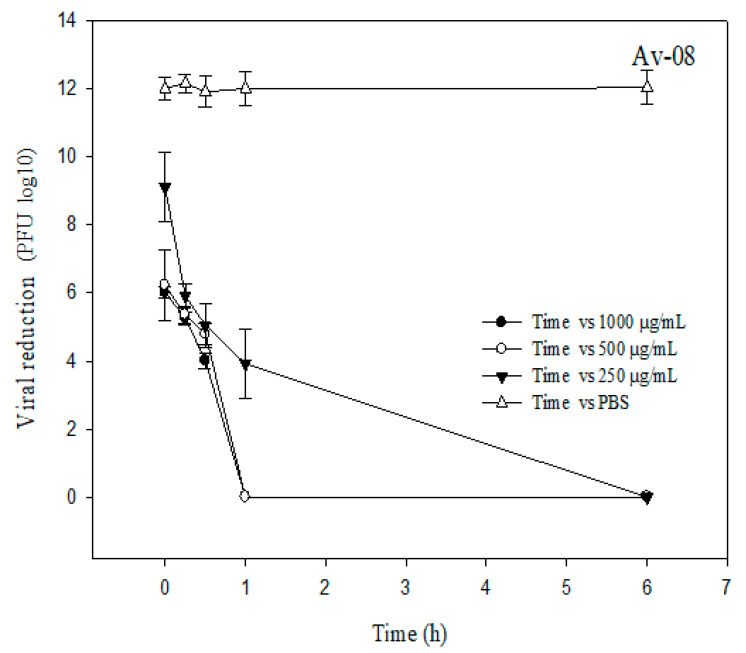
Reduction of Av-08 (PFU/mL log10) after treatment with AEE for various contact times (0, 15, 30, 60 and 360 min) at three concentrations (250, 500 and 1000 µg/mL). The vertical error bars represent the standard deviation (SD). Phosphate-buffered saline (PBS) is the control.

**Figure 2:**
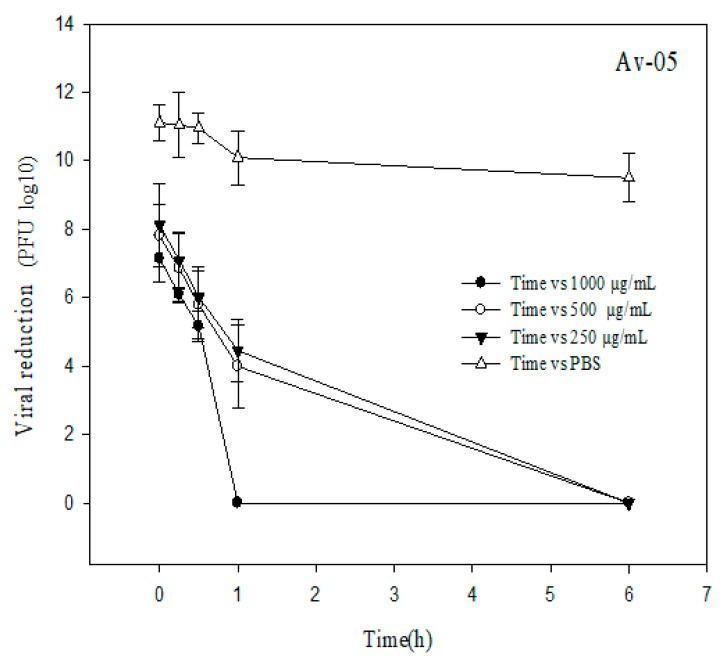
Reduction of Av-05 (PFU/mL log10) after treatment with AEE for various contact times (0, 15, 30, 60 and 360 min) at three concentrations (250, 500 and 1000 µg/mL). The vertical error bars represent the standard deviation (SD). Phosphate-buffered saline (PBS) is the control.

Figure 1 and Figure 2 show the log10 reduction in bacteriophages Av-05 and Av-08, respectively. The highest reduction in the phages was observed in response to 1 mg/mL AEE and with a contact time longer than 30 min, and with values of 7 and 4 log10 PFU/mL recorded for Av-05 and Av-08, respectively. The antiviral effect of the extract at a concentration of 0.25 mg/mL during the fourth contact time (60 min) showed a reduction of 9 and 5 log10 PFU/mL for Av-05 and Av-08, respectively, whereas, for the longest contact time (360 min), we observed a total reduction in coliphages. The control condition in phosphate-buffered saline (PBS) showed no significant reduction in viral titer during treatments. 

The antiviral activity of the extract could also be observed at a concentration of 0.5 mg/mL, which reduced the Av-05 and Av-08 phage titers by 4 and 6 log10 PFU/mL, respectively, at the first contact time (0 min). We observed that the antiviral activity of AEE was dose- and time-dependent, as increasing concentration and contact time increased viral inhibition.

## 3. Discussion

Most of the phenolic compounds of the soursop leaf extracts evaluated in the present study corresponded to rutin and naringenin, while vanillin, gallic acid and eugenol were minor fractions (Table 1). Rutin (the rhamnoglucoside of the flavonoid quercetin) was the principal compound in *A. muricata* leaves. This result has been determined in previous studies, such as that of Son et al. [13], who reported high concentrations of rutin in *A. muricata* leaves, even higher than other sources. In an investigation by Calzada et al. [14], among purified rutin and other metabolites from *A. muricata* leaf fractions, rutin showed significant antihyperglycemic activity. The other compounds found in the present study have been extracted from the stems, leaves, roots and fruits of other Annonaceae, including vanillin [15], gallic acid [16], eugenol [17] and naringenin [18].

The antioxidant capacity of the extracts is influenced by the pH and chemical nature of the solvents used in the extraction, as less polar solvents show a greater capacity to dissolve bioactive antioxidant substances. The antioxidant activity of AE by DPPH test was the lowest among all of the samples included in this study. This low antioxidant capacity could be related to the low contents of phytochemicals such as phenolics and flavonoids in these extracts (Table 1). In previous research by Kuskoski et al. [19] that evaluated *A. muricata* pulp using the DPPH radical assay, the values of antioxidant activity were different than those found in the present study. This is mainly because the leaves are more metabolically active than the fruit, and their secondary metabolism produces antioxidant compounds as a response to environmental factors. On the other hand, it is observed that in the antioxidant capacity for AEE measured by DPPH and ABTS, it was higher than AE. These results indicated that the phenolic compounds in the AEE (Table 1) contributed positively to their antioxidant capacity by reducing the levels of free radicals.

The protective capacity of soursop leaves evaluated in the present study (Table 3) could be explained by the nature of the phenolic compounds in the leaves. These substances could be responsible for their antihemolytic effect as they not only stabilize free radicals, but also increase the resistance of erythrocytes to oxidative stress [20]. The effect observed in human erythrocytes may be because the mixture of ethanol and acetic acid in AEE increases the ability to hydrolyze bonds, favoring the extraction and availability of antioxidant compounds, such as polyphenolic substances. Additionally, the antioxidant compounds present in the extracts could donate one or more electrons to neutralize the AAPH radical by inhibiting hemolysis [12]. The results for the antihemolytic effect of soursop leaf extracts were similar to those of Cyboran et al. [21], who analyzed changes in pig erythrocytes induced by extracts of currant, strawberry and apple leaves, and they showed that the polyphenolic substances present in the extracts had a positive effect on erythrocytes. This effect was attributed to the capacity of the extracts to interact with the outer layer of the lipid membrane, permeating the hydrophilic part where they cause changes in the packaging arrangement of the polar heads of the lipids. This makes it more elastic and resistant to changes in pressure of the osmotic environment and, therefore, also more resistant to damage caused by oxidative stress due to the correlation between polyphenol content and antioxidant effects. Flavonoids could be related to the protection of cell membranes because they interrupt the interaction of phospholipid components and inhibit their oxidation, thereby protecting them from damage caused by oxidizing molecules [6].

In the antiviral study, we observed a dose-dependent behavior. As the dose and contact time with AEE is increased, viral replication decreases after an hour of contact time. Likewise, the antiviral efficacy displayed in the bacteriophages, used as human viral surrogates (Figure 1 and Figure 2), could be explained with the results in the phenolic profile (Table 1), where rutin is the main component. Rutin is an effective inhibitor of dihydrofolate reductase and shows antiviral, anticancer, anti-inflammatory and heart disease protective activities [22]. Rutin interacts with multiple viral proteins and is safe to consume [23]. Even recent in vivo and in silico studies have shown that rutin is an excellent candidate to eliminate viral replication of SARS-CoV and SARS-CoV-2 (COVID-19), respectively; for example, Elmi et al. [24] suggest that rutin acts on angiotensin-converting enzyme 2 (ACE 2), which is the receptor that binds to the spike glycoprotein of SARS-CoV-2 that facilitates membrane fusion and viral infection. Rutin could potentially inhibit ACE 2 to suppress the entry of SARS-CoV-2. The present study found high levels of flavonoid glycosides, with naringenin being the second most abundant component in AEE. A review by Ling et al. [25] reported that quercetin and naringenin in the antiviral Lianhua Qingwen formulation might target the angiotensin-converting enzyme, which could be one of the direct targets to inhibit SARS-CoV-2, and protect target organs through the renin–angiotensin pathway. Another compound found in the present study is gallic acid, and there is evidence that this compound can inhibit human immunodeficiency virus, hepatitis C virus, herpes simplex virus and *Haemophilus influenzae* A and B [26]. This is supported by a study by Hinkov et al. [27], who reported that quercetin and vanillin present antiviral activity against herpesviruses. By last, AEE contains eugenol, which shows broad antiviral, antimicrobial, antifungal and anti-inflammatory activity, including against the Ebola virus [28].

Although the exact mechanisms of action of AE and AEE on biological activity and the different variables examined in this study could not be established, a number of previous scientific publications have stated that polyphenolic compounds and other compounds, as well as acetogenins, possess properties as antioxidant or antivirals. Astirin et al. [29] analyzed various extracts of soursop leaves to induce apoptosis in cancer cells caused by the human papillomavirus (HPV) and herpes simplex, observing that acetogenins and ellagic acid can induce cellular apoptosis and antivirals effects, respectively. They concluded that the antiviral activity could be due to acetogenins, which are phytochemicals of *A. muricata* L. leaves that have potent antiviral activity. Although these substances were not analyzed in the present study, they could also be involved in the antiviral activity of the analyzed extracts. Therefore, it is not unreasonable to speculate that some of the plant chemicals mentioned above are probably responsible for the antiviral activity as well as the antioxidant property of AEE especially. Furthermore, AEE could be used for pharmaceutical purposes because it has been demonstrated that this kind of extract is considered safe [6,30].

## 4. Materials and Methods

### 4.1. Reagents

Potassium persulfate, 2,2-azino-bis (3-ethylbenzothiazoline-6-sulfonic acid) diammonium salt (ABTS), 2,2-diphenyl-1-picrylhydrazyl (DPPH), 6-hydroxy-2,5,7,8-tetramethylchroman-2-carboxylic acid (Trolox), AAPH radical, polyphenol standards, gallic acid, ferulic acid, rutin, naringenin, vanillin, eugenol, quercetin, chlorogenic acid and caffeic acid were obtained from Sigma-Aldrich (St. Louis, MO, USA). Water, methanol, acetic acid, acetonitrile (high-performance liquid chromatography (HPLC) grade) and dimethyl sulfoxide (DMSO) were obtained from J.T. Baker (Baker-Mallinckrodt, Mexico City, Mexico).

### 4.2. Vegetal Material

The leaves of *A. muricata* L. were obtained from 9-year-old tree crops in San Blas, Nayarit, Mexico. The material was dried at 45 °C for 24 h in a Blinder oven (model ED 115) and then powdered and passed through a sieve (number 20). The AEE was obtained using 10 g of dried leaves mixed with 150 mL of 90% ethanol and 10% acetic acid (9:1), which was macerated with agitation in complete darkness for 72 h. The extract was concentrated in a rotatory evaporator (RE301; Yamato, Japan). For the AE, 10 g of dried leaves were mixed with 150 mL of distilled water and boiled for 5 min; then the extract was filtered and concentrated as previously described.

### 4.3. UPLC Analysis

The chromatographic analysis was performed according to Hernández et al. [31] using Waters UPLC analytical system (Waters Corp., Singapore) equipped with a diode array UV detector. An Acquity UPLC BEH C18 1.7-μm column (2.1 × 50 mm) was used. Likewise, three mobile phases were used to promote the separation of the phenolic compounds: (A) distilled water with 0.1% acetic acid, (B) methanol and (C) HPLC-grade acetonitrile. The separation had a total running time of 14 min at a temperature of 35 °C and 20 °C for the column and sample, respectively, at a wavelength of 280 nm AC. An elution gradient was used starting with 90% A, 5% B and 5% C; changing the ratio at 6 min to 76% A, 12% B and 12% C; at 11 min to 36% A, 32% B and 32% C; and then finally changing back to the initial gradient at 12 min until the run was finished. The technical details of this study such as reproducibility, chromatograms, absorption spectra and linearity are shown in the Supplementary Material.

### 4.4. DPPH Assay

The antioxidant capacity was measured according to Moein and Moein [32]. A 280 μL aliquot of the DPPH radical solution (0.025 mg/mL in methanol) was mixed with 20 μL of the extract to be evaluated and kept to stand in the dark for 30 min. Absorbance was read at 490 nm in a microplate reader (iMark Microplate Reader; BioRad, Chicago, IL, USA). The antioxidant activity was calculated using a Trolox calibration curve. The total equivalent antioxidant capacity (TEAC) value was expressed as Trolox equivalents (mmol TE) per gram.

### 4.5. ABTS Assay

The assay was developed according to Re et al. [33]. The radical was prepared with 19.2 mg of ABTS dissolved in 5 mL of water and 88 μL of potassium persulfate (0.139 mM). This solution was incubated in the dark at room temperature for 16 h. The stock solution was prepared by mixing 1 mL of the ABTS radical with 88 mL of ethanol. A 500 μL aliquot was taken from this incubated solution and diluted in ethanol to adjust the absorbance to 0.7 ± 0.02 at 750 nm. Finally, 295 μL of the radical and 5 μL of the extract were placed in a microplaque, and were allowed to stand in the dark for 7 min. The antioxidant activity was calculated by comparison to a Trolox calibration curve. The TEAC value was expressed as Trolox equivalents (mmol TE) per gram. 

### 4.6. Hemolysis Assays in Human Erythrocytes

Hemolysis was induced by AAPH radicals according to Lu et al. [34]. Briefly, the erythrocytes were washed three times with PBS (pH 7.4). After washing, a 5% suspension of human erythrocytes in PBS was prepared. For the assay, 50 μL of the erythrocyte suspension, 50 μL of the extract and 200 μL of AAPH were mixed and incubated at 37 °C in a shaking bath (30 rpm) for 3 h. A similar reaction mixture was prepared without extract as a control. After incubation, 1 mL of PBS was added and the mixture was centrifuged at 3500 rpm for 10 min, and then the absorbance was measured at 540 nm in a microplate reader (iMark; BioRad, Chicago, IL, USA). The result was expressed as a percentage of inhibition, calculated using the following equation: [(absorbance control − absorbance final)/absorbance control × 100].

### 4.7. Bacteriophage Propagation

The bacteriophages Av-05 y Av-08 were obtained from the National Laboratory for Research in Food Safety of the Centro de Investigación en Alimentación y Desarrollo (CIAD) in Culiacan, Sinaloa, Mexico. For their propagation, 100 μL of the purified bacteriophage solution was mixed with 1 mL of *Escherichia coli* O157 with 3 mL of 0.4% tryptic soy agar (TSB) agarose. This suspension was poured into tryptic soy agar (TSA) and incubated at 37 °C for 18–24 h. After incubation, the buffer solution was added and shaken for 2 h. The surface was recovered and centrifuged at 10,000× *g* at 4 °C for 15 min. The supernatant was filtered through a nitrocellulose membrane (Whatman, Marlborough, MA USA) with 0.45-μm pore diameter, and then centrifuged at 10,000× *g* at 4 °C for 2 h [35].

### 4.8. Antiviral Assay

The extract was dissolved in sterilized distilled water and filtered through a nitrocellulose membrane with a 0.45-μm pore diameter to ensure sterility. The concentrations tested were 0.25, 0.50 and 1 mg/mL, with each concentration evaluated in duplicate. In this assay, a 100 μL aliquot of the phage was challenged with 3 mL of the extract. Five contact times were evaluated: 0, 15, 30, 60 and 360 min. Then, serial dilutions of 10^−2^ to 10^−10^ were made in PBS. To tubes containing 3 mL of 0.4% TSB agarose, 50 μL of each dilution and 500 μL of the host cell were added. The mixtures were gently shaken and deposited on Petri dishes with TSA. The Petri dishes were incubated at 37 °C for 24 h, and the plaque-forming units (PFU) per milliliter were measured for each sample. The effects were determined separately. A control consisted of 50 μL of the bacteriophage with 1 mL of *E. coli* O157 without the soursop extracts.

### 4.9. Data Analysis

To obtain quantitative information on the antioxidant capacity of the extracts, a completely randomized experimental design was used. Three replicates were used and a *p*-value < 0.001 was considered significant.

The analysis used to evaluate the effect of extracts on bacteriophages was randomized considering two factors: the concentration analyzed (0.25, 0.50 or 1 mg/mL) and the contact time with the phage (0, 15, 30, 60 or 360 min). A viral reduction was expressed as the log10 phage titer. Treatments were performed in duplicate, with three replicates in each. In both designs, Statgraphic plus 5.1 software was used to perform the analysis of variance. 

## 5. Conclusions

The AEE extract obtained from *A. muricata* leaves may represent an alternative pharmaceutical application due to its antioxidant, antiviral and antihemolytic activities, as demonstrated in the present study. The phenolic profile includes several compounds, including rutin, the main flavonoid due to its high concentration in the extracts AE and EEE, and it is possibly responsible for the activities evaluated. However, the antioxidant and antiviral effect of this compound must be further analyzed to establish its action mechanism.

## Figures and Tables

**Table 1:** Detection and quantification of the phenolic composition of *Annona muricata* L., considering both extracts.

	Phenolic Compounds	mg/g	
		AE	AEE
1	Gallic acid (3,4,5-trihydroxybenzoic acid)	ND	3.13 ± 0.14
2	Rutin (quercetin 3-*O*-rutinoside)	1.20 ± 0.06	6.52 ± 0.59
3	Naringenin (5,7-dihydroxy-(2-4-hydroxyphenyl) chroman-4-one)	ND	5.22 ± 0.75
4	Vanillin (4-hydroxy-3-methoxybenzaldehyde)	ND	3.60 ± 0.17
5	Eugenol (4-allyl-2-methoxyphenol)	ND	1.40 ± 0.04

Results are expressed as mean ± standard deviation (*n* = 3) and correspond to the milligrams per gram of dry weight. ND, not detected. Aqueous Extract (AE), Acidified Ethanolic Extract (AEE).

**Table 2:** Antioxidant effect of *Annona muricata* L. extracts measured by DPPH and ABTS assay.

Extracts	DPPH (mmol TE/g)	ABTS (mmol TE/g)
AE	2.97 ± 0.40 ^a^	17.93 ± 0.31 ^b^
AEE	23.61 ± 0.42 ^b^	24.91 ± 0.16 ^b^

Results are expressed as mean ± standard deviation of three determinations. Means with different letters within a column are significantly different (*p* < 0.05).

**Table 3:** Protective effect of *Annona muricata* L. extracts on human erythrocytes.

Extracts	Antihemolytic Effect (%)
AE	34.16 ± 0.13 ^a^
AEE	51.21 ± 0.36 ^b^

Results are expressed as mean ± standard deviation (*n* = 3). Means with different letters within a column are significantly different (*p* < 0.05).

## Supplementary Materials

The following are available online at https://www.mdpi.com/2223-7747/9/12/1650/s1. Table S1: Reproducibility test results; Figure S1: UPLC chromatogram of phenolic compound standards analyzed at 280 nm; Figure S2: UPLC chromatogram of phenolic compounds present in AEE; Figure S3: UPLC chromatogram of phenolic compounds present in AE. Peaks; Figure S4: Absorption spectra/retention time of rutin compound compared to samples.
