# Supplementary material for: Antiviral, Antioxidant, and Antihemolytic Effect of Annona muricata L. Leaves Extracts
Source: Plants (Basel). 2020 Nov 26;9(12):1650. doi: 10.3390/plants9121650 (PMC7760998; doi:10.3390/plants9121650)
Supplement: Supplementary file 1 [file plants-09-01650-s001.pdf]

Supplementary material

Validation

Reproducibility

The reproducibility was evaluated as the relative standard deviation (RSD) of peak area of 12 standard solutions. Through the analyzes, in all the cases, RSD of the peak areas was ≤4.5% (Table S1).

Table S1. Reproducibility test results

	**Standard**	**Retention time (min)**
1	Gallic acid	0.60±0.02
2	Resorcinol	1.15±0.02
3	Chlorogenic acid	1.955±0.045
4	Caffeic acid	2.475±0.035
5	Vanillin	3.455±0.035
6	Coumaric acid	4.175±0.035
7	Ferulic acid	5.1±0.03
8	Rutin	6.677±0.025
9	Naringenin	8.80±0.02
10	Trans cinnamic acid	9.325±0.015
11	Kaempferol	9.615±0.015
12	Eugenol	9.89±0.20

A bibliographic survey was carried out to study the phenolic profile. 12 compounds were selected as shown in the Figure S1, where the retention times of each standard are detailed, of them, only 5 were detected as shown in Figure S2, and one in Figure S3.

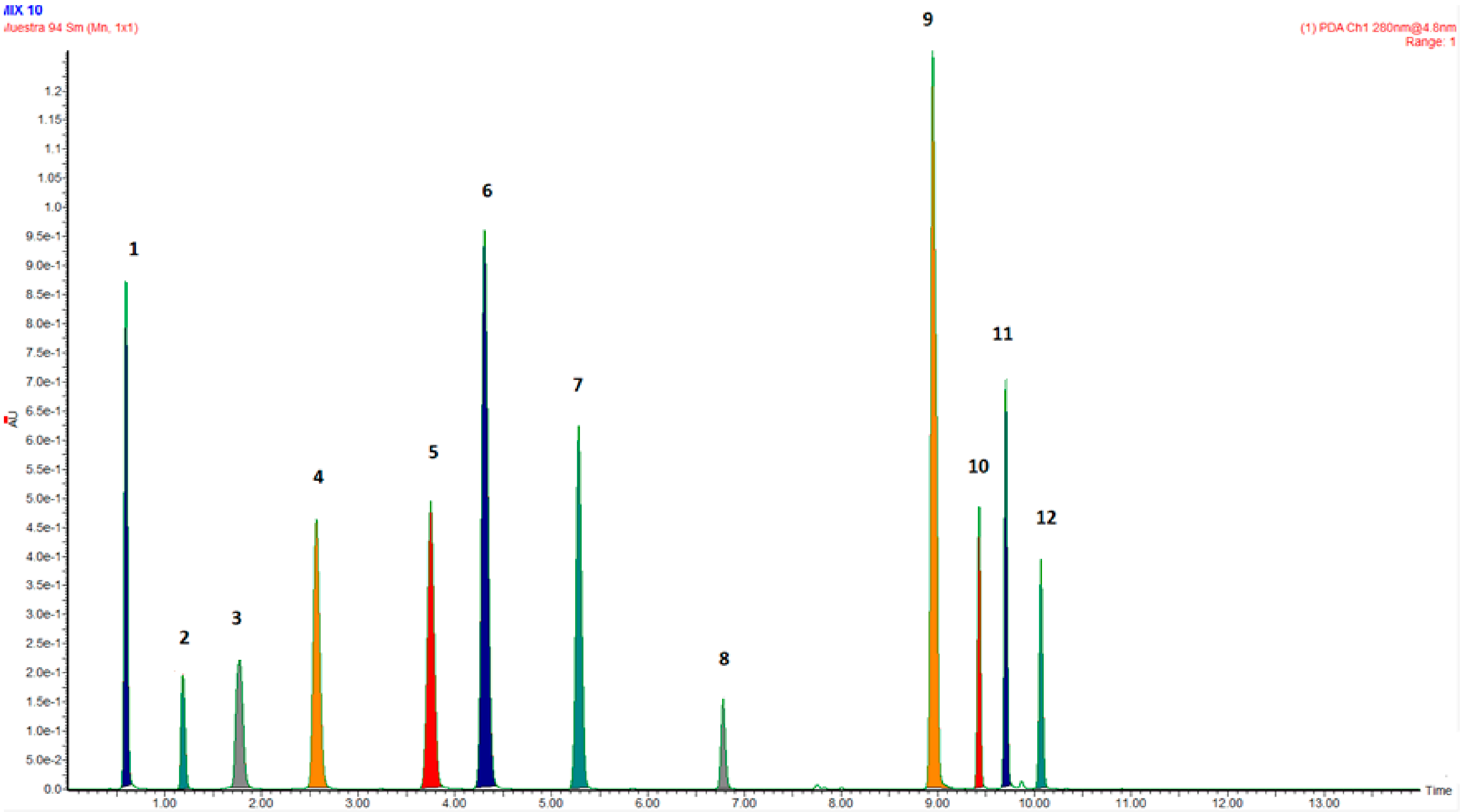

Figure S1. UPLC chromatogram of phenolic compound standards analyzed at 280 nm [y axis = intensity (absorbance unit, AU); x axis = retention time (min). Peaks: 1, gallic acid; 2, resorcinol; 3, chlorogenic acid; 4, caffeic acid; 5, vanillin; 6, coumaric acid; 7, ferulic acid; 8, rutin; 9, naringenin; 10, quercetin;11, kaempferol and 12, eugenol.

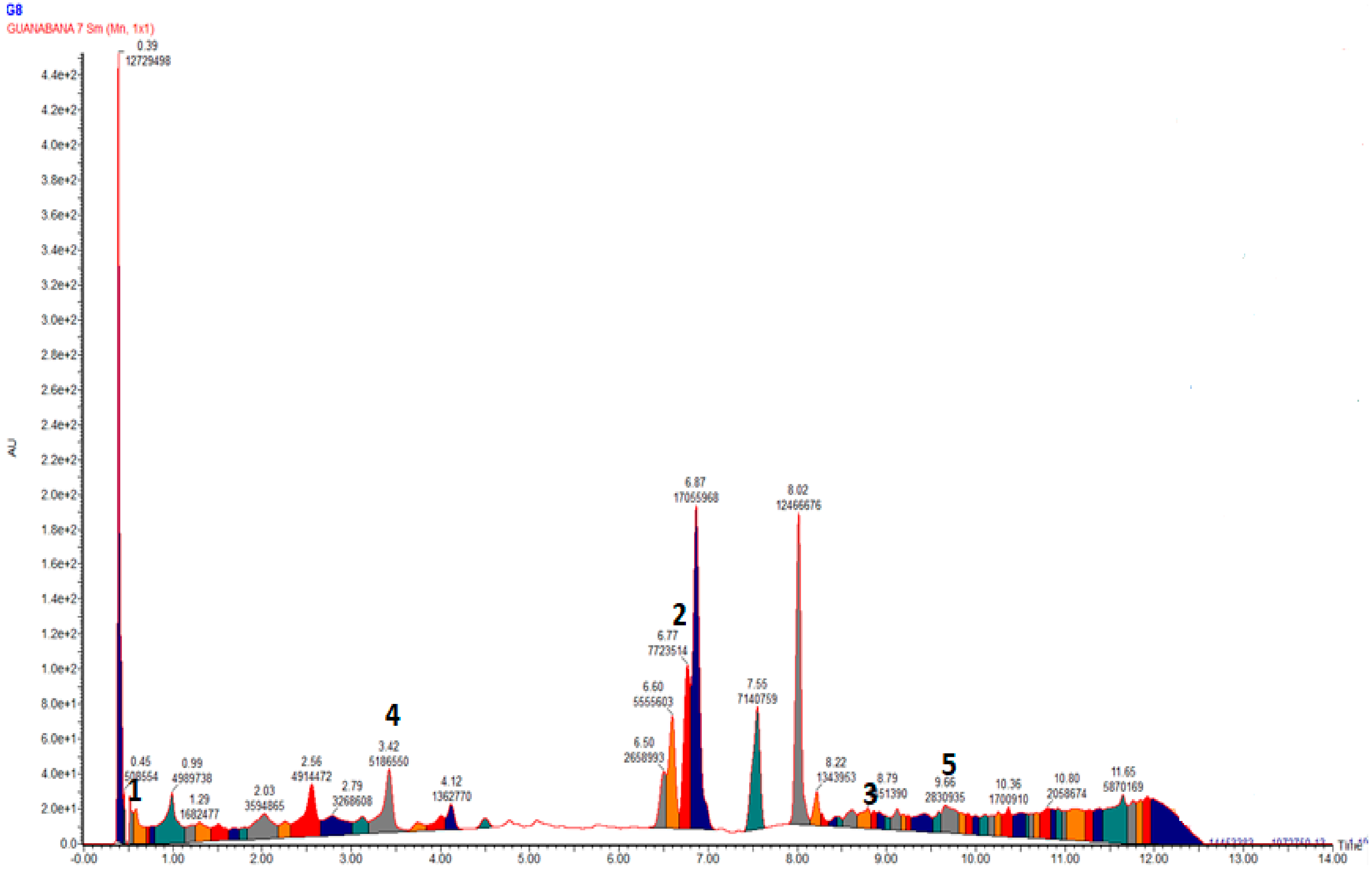

Figure S2. UPLC chromatogram of phenolic compounds present in AEE. Peaks: (1) gallic acid, (2) rutin, (3) naringenin, (4) vanilin, and (5) eugenol.

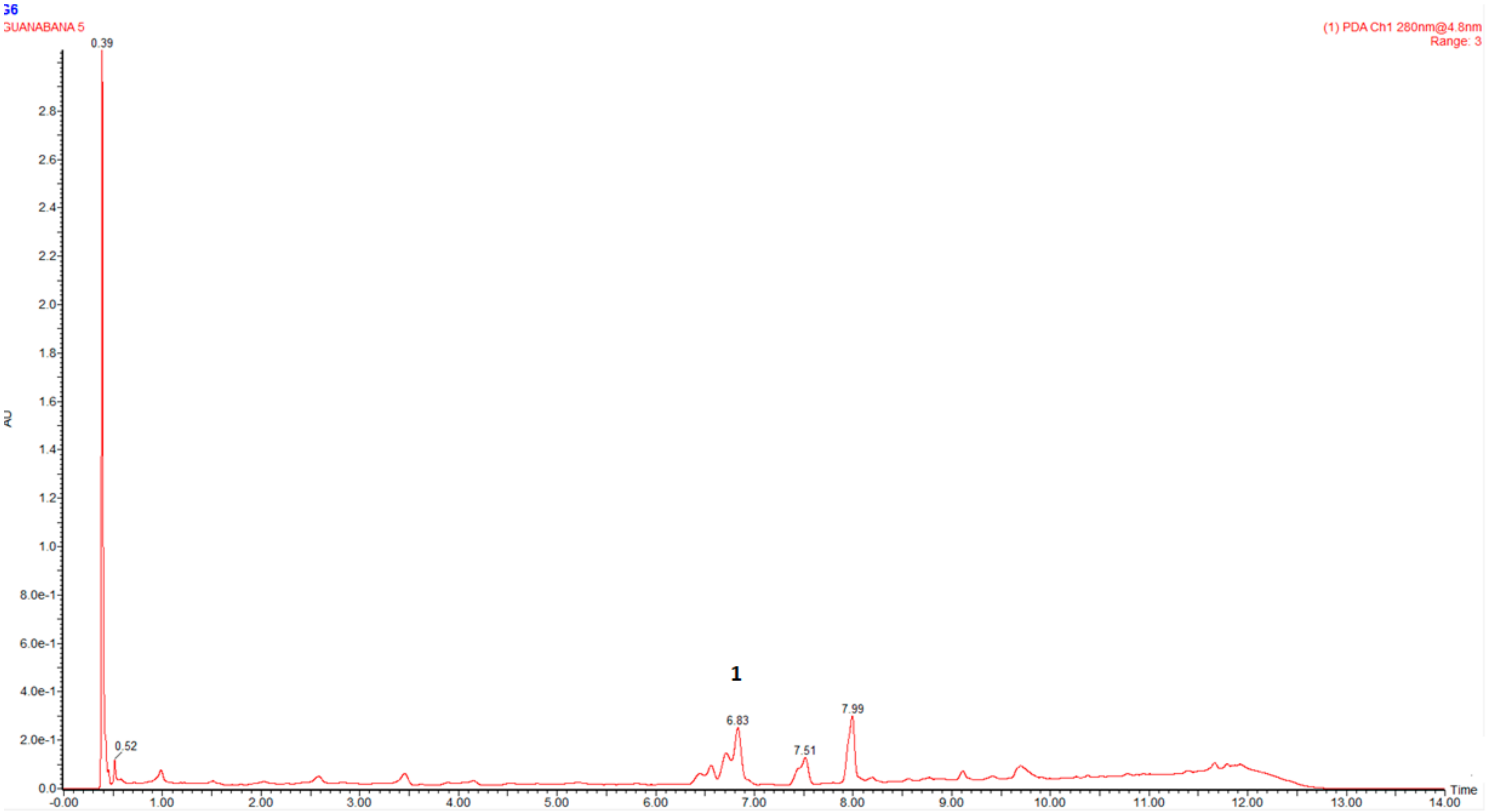

Figure S3. UPLC chromatogram of phenolic compounds present in AE. Peaks: (1) Rutin.

Linearity

Linearity was calculated by analyzing four different standard solutions. The calibration curves were created by plotting the response, under-curve area, versus the concentration used. The calibration curve showed the following values: slope 86.136, intercept -105.74, and correlation coefficient .99 for rutin, gallic acid, naringenin, vanillin and eugenol. The LOQ was 0.2 µg/mL.

Detection

For the identification of the compounds, a comparison was made of the absorption spectrum generated by integrating the area under the curve in the retention time detected in the standard. As an example, Figure S4 shows the absorption spectra of the routine standard (the main component detected of this study) and the absorption spectra of the samples at different concentrations.

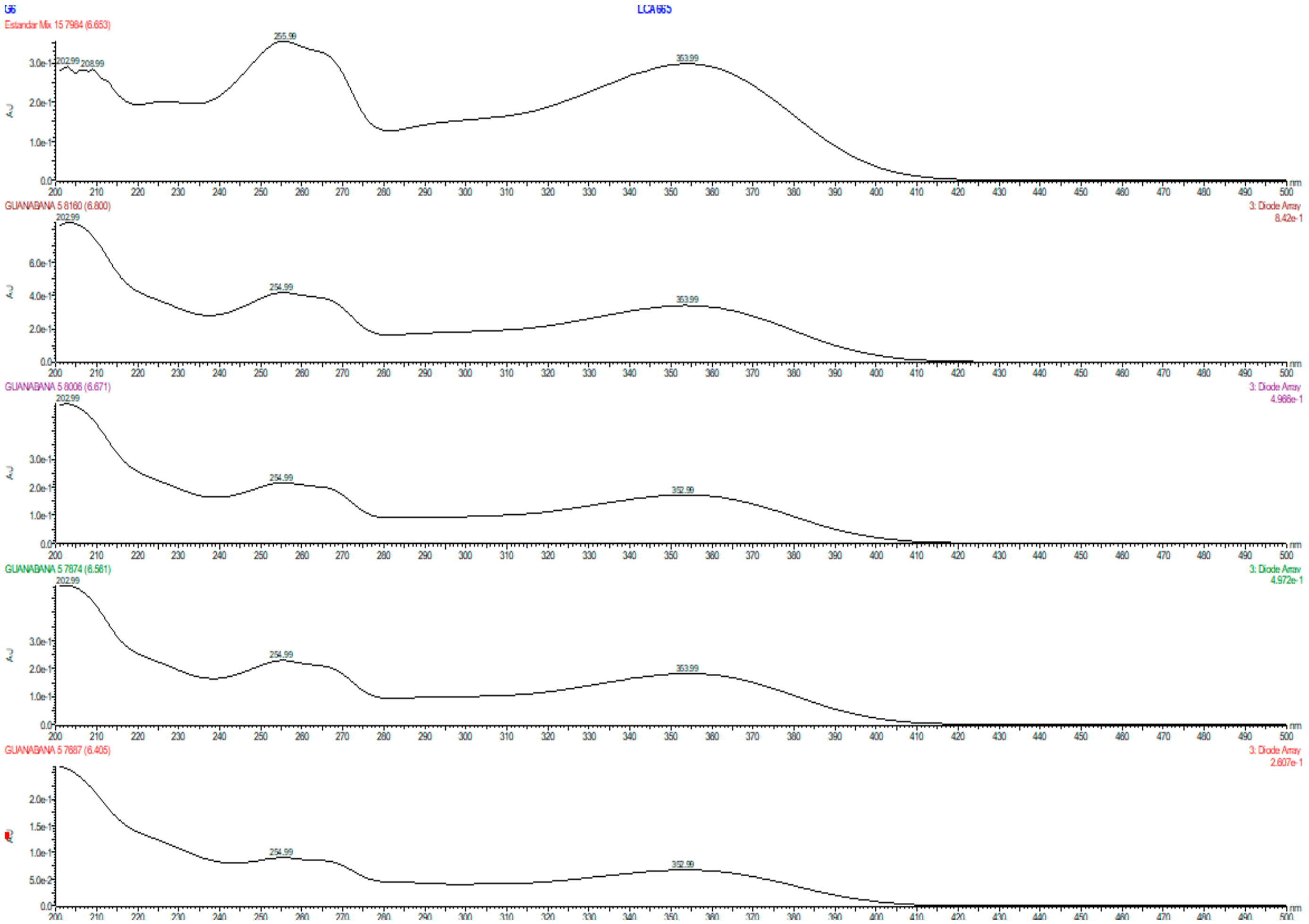

Figure S4. Absorption spectra/retention time of rutin compound compared to samples.
